# Clinical Applications of Laser Technology: Laser Balloon Ablation in the Management of Atrial Fibrillation

**DOI:** 10.3390/mi12020188

**Published:** 2021-02-12

**Authors:** Jamario R. Skeete, Jeanne M. Du-Fay-de-Lavallaz, David Kenigsberg, Carlos Macias, Jeffrey R. Winterfield, Parikshit S. Sharma, Richard G. Trohman, Henry D. Huang

**Affiliations:** 1 Division of Cardiology, Rush University Medical Center, Chicago, IL 60612, USA; Jamario_R_Skeete@rush.edu (J.R.S.); Parikshit_S_Sharma@rush.edu (P.S.S.); Richard_Trohman@rush.edu (R.G.T.); 2 Department of Internal Medicine, University Hospital Basel, 4031 Basel, Switzerland; Jeanne_M_Du-Fay-de-Lavallaz@rush.edu; 3 Division of Cardiology, Nova Southeastern University, Fort Lauderdale-Davie, FL 33314, USA; dkenigsberg@flahrs.com; 4 Section of Cardiology, Ronald Reagan University of California Los Angeles Medical Center, Los Angeles, CA 90095, USA; CarlosMacias@mednet.ucla.edu; 5 Division of Cardiology, Medical University of South Carolina, Charleston, SC 29425, USA; winterfj@musc.edu

**Keywords:** atrial fibrillation, atrial fibrillation ablation, pulmonary vein isolation, cryoballoon ablation, radiofrequency ablation, laser balloon ablation, catheter ablation, rhythm control, fluoroless

## Abstract

Catheter-based ablation techniques have a well-established role in atrial fibrillation (AF) management. The prevalence and impact of AF is increasing globally, thus mandating an emphasis on improving ablation techniques through innovation. One key area of ongoing evolution in this field is the use of laser energy to perform pulmonary vein isolation during AF catheter ablation. While laser use is not as widespread as other ablation techniques, such as radiofrequency ablation and cryoballoon ablation, advancements in product design and procedural protocols have demonstrated laser balloon ablation to be equally safe and effective compared to these other modalities. Additionally, strategies to improve procedural efficiency and decrease radiation exposure through low fluoroscopy protocols make this technology an increasingly promising and exciting option.

## 1. Introduction

Practical applications of laser technology are widespread in multiple areas of medicine. In cardiac electrophysiology, there has been growing use of this energy source for extraction of pacemaker and implantable cardioverter defibrillator (ICD) leads and atrial fibrillation (AF) catheter ablation [1,2].

Given the widespread nature of AF and the morbidity/mortality associated with this disease, several approaches have been employed to decrease disease burden [3]. Restoration of normal sinus rhythm via catheter-based pulmonary vein antral isolation (PVAI) has become an established strategy for symptomatic AF patients or those refractory to antiarrhythmic therapy [4]. More recently, utility of this invasive rhythm control strategy has been extended to early in the disease course as an effective means to improve patient outcomes [5,6]

For over 20 years, the mainstay of catheter-based ablation of paroxysmal and persistent AF involved circumferential electrical isolation of the source of electrical triggers (the pulmonary veins), from the left atrium (LA) induction of point-by-point coagulation necrosis of the ostium of the pulmonary veins using radiofrequency energy. While highly effective, drawbacks to radiofrequency ablation (RFA) techniques exist. These drawbacks include a steep operator learning curve; prolonged procedural times; as well as potential complications such as thrombus formation/embolization, phrenic nerve paralysis, cardiac tamponade, atrio-esophageal fistulae, and pulmonary vein stenosis. In addition to these limitations, recurrence of AF may occur in as much as 50% of persons with persistent AF within the first 18 months of the initial RFA procedure [7]. As a result, techniques utilizing alternate energy sources including cryotherapy, and more recently laser and pulsed electric fields have been explored with clinical efficacy comparable to RFA with similar procedural and side effect profiles [8,9].

In this review, we explore the evolving technique of laser balloon ablation (LBA) for management of paroxysmal and persistent AF. The biomechanics of this system are thoroughly explained and the clinical applications of LBA compared to alternate existing ablation strategies are discussed in detail.

## 2. Biophysics and Biomechanics of Laser Balloon Ablation

**Figure 1:**
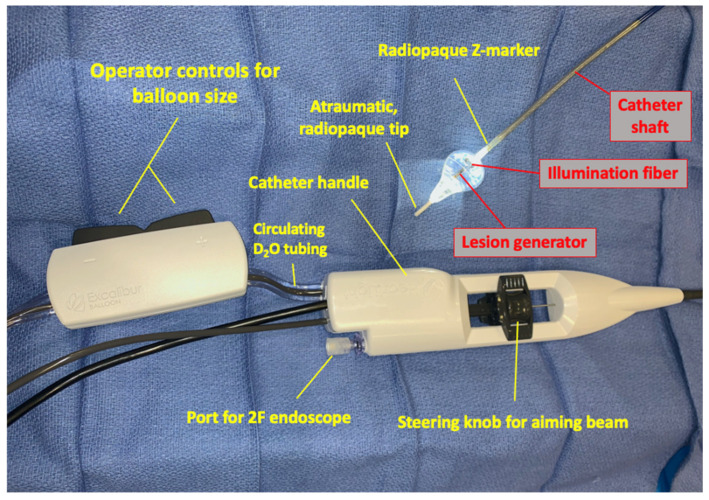
Components of HeartLight X3 3rd-generation laser ablation system shown.

The endoscopic laser ablation system (Heartlight^®^, CardioFocus, Inc., Marlborough, MA, USA) is the only commercially available device utilizing laser technology for PVAI AF management. This device, currently in its third generation, consists of a catheter shaft (12 French) with an inflatable compliant balloon (maximum expandable diameter 37 mm) and a flexible non-traumatic distal tip. Within the catheter is an optical fiber with a custom laser energy reflector called a lesion generator at the distal end which delivers the 980 nm laser energy, all of which is housed in the shaft inside the inflatable compliant balloon. At the operator end is a console which allows control of the power emitted and duration of laser application as well as connections to the light source and direct endoscopic visual display system (Figure 1). As an alternative to electropotential measuring capabilities (such as those utilized in other RF ablation systems), navigation and identification of the site of lesion creation are permitted via direct tissue visualization using an embedded 2French endoscope. It is worthwhile to note that while the small profile of the endoscope allows for visualization of the pulmonary veins while using the catheter, this comes at the expense of diminished image quality, particularly when compared to larger endoscopes used in other medical procedures, such as laryngoscopy. The size and compliance of the balloon itself can be directly adjusted by the operator via a handheld controller to accommodate pulmonary vein sizes from 8 mm to as large as 40 mm at any time during the procedure, which is a unique feature of the technology [8].

The laser diode emits energy perpendicular to the housing catheter via a lesion generator which, in this current device generation, covers an arc of 30 degrees and has an adjustable aiming point. In this system, the lesion generator is housed within the compliant balloon which uses deuterium oxide (D_2_O) as the inflation fluid and laser conduction fluid [10]. Deuterium oxide is used as opposed to saline or other fluids as the inflation medium to eliminate self-heating of the balloon itself [11]. Adjustments of the energy delivery can be made ranging from 5.5 to 15 W, with duration of laser application being 20 or 30 s, or continuously at higher doses based on the operator preference.

The basis for the use of laser as the energy source in LBA stems from the knowledge that specific structural changes may take place when light energy is applied to cardiac tissue [2]. These interactions, which are impacted by both laser and tissue properties, include photochemical (inactivation of cell function without increases in tissue temperature), photothermal (light energy is converted to heat by the tissues), and photoplasmal (tissue rupture occurs through the production of high elective fields and shock waves) reactions. The specific impact of the application of the laser source depends the interplay between laser properties (such as the laser wavelength and duration of application), and tissue factors (such as tissue density and water composition) [12].

For the purpose of LBA, the 980 nm produces transmural tissue disruption primarily via photothermal laser–tissue interactions, which has been observed on both histological and gross pathologic specimen in animal studies [13].

## 3. Procedural Access

As is the case with other catheter-based AF ablation techniques, this modality utilizes a percutaneous femoral vein access and ideally an inferior-posterior placed atrial transseptal puncture under the guidance of fluoroscopy or intracardiac echocardiography (ICE). The locations of the pulmonary veins are by convention, identified via ICE, 3D electroanatomical mapping, or fluoroscopic guidance [2].

## 4. Lesion Generation

**Figure 2:**
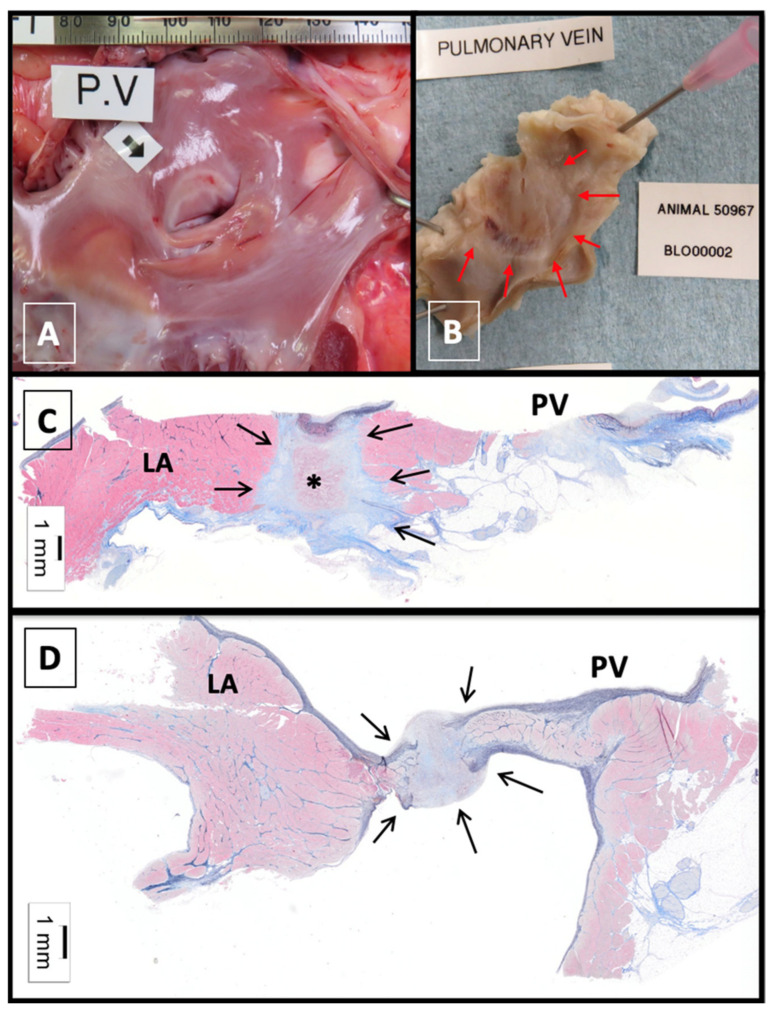
Study examining safety and efficacy of Cardiofocus X3 catheter in a swine model simulating isolation of right superior pulmonary vein in clinical use. Ablation performed with energy delivery in Rapid Mode at maximum 15 Watt power level around the pulmonary vein’s entire circumference following inflation of the balloon of the ablation catheter just to the point at which the balloon has no wrinkles. Panels show chronic necropsy and histologic findings after ablation using HeartLight X3 endoscopic ablation system in the vivo swine model, Day 34. (**A**) Ex vivo sample at necropsy after triphenyl-tetrazolium chloride staining. Black arrow indicates circumferential ablation at the pulmonary vein ostium. Treated area is sharply demarcated and pale without thrombus or evidence of injury at more distant tissue locations. (**B**) Formalin-fixed (unstained) sample with pulmonary vein ostium opened to expose well-demarcated treatment zone (red arrows). (**C**,**D**) Hematoxylin and eosin and Masson’s trichrome stained sample, Day 34, Pulmonary vein. Arrows indicate well-demarcated, transmural focus of ablation which contains residual focus of necrotic myocardial/medial tissue at center (asterisk). Images used with permission from Cardiofocus.

**Figure 3:**
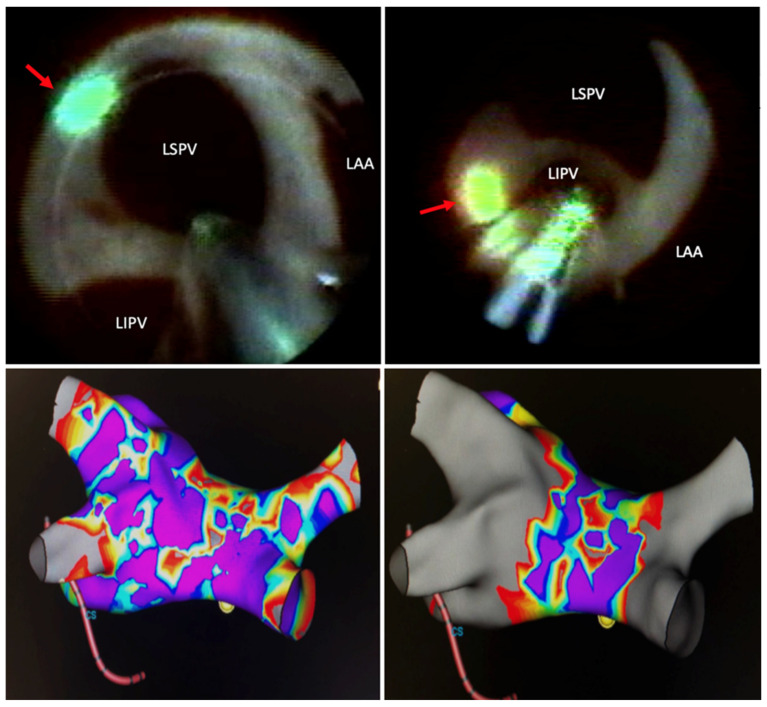
LSPV: left superior pulmonary vein; LIPV: left inferior pulmonary vein; LAA: left atrial appendage; red arrow: green-colored aiming beam of lesion generator. Top panels show endoscopic views during laser ablation of the LSPV and LIPV. Bottom panels show pre-ablation voltage map of left atrium and pulmonary veins (**left**) and post-ablation voltage map wide area antral isolation of the pulmonary veins (**right**).

The basic principle of PVAI is to electrically separate conduction from the muscular sleeves of the pulmonary veins (from where spontaneous, irregular electrical activity is harbored) that triggers AF, from the remaining atrial tissue. In LBA, electrical isolation is produced by a continuous beam of photonic energy focused in sequential or continuous fashion to the antral surface proximal to the pulmonary veins. This mechanism seeks to improve on existing deficiencies with radiofrequency ablation, in which the disruption of the endothelium may act as a substrate for clot formation and subsequent thromboembolic events (Figure 2). Visually guided LBA also allows for “stitching” of sequential energy applications to avoid gaps in necrotic lesions to lessen the risk of chronic reconnection of pulmonary veins or reentrant atrial arrhythmias post-ablation (Figure 3). Additionally, this method seeks to overcome ablation at the ostium of the pulmonary veins, which had been associated with increased rates of pulmonary vein stenosis [13].

Compared to RFA, in which there is direct endothelial injury, the biomechanics of LBA varies, in that photonic energy results in volumetric heating. Due to the variations in water content in the different layers of heart tissue as well as thermal mechanics, peak temperature achieved is subsurface (and not on the surface as is the case in RFA), thereby limiting the likelihood of endothelial damage and avoiding thrombus formation [10]. Theoretically, this should translate to lower rates of pulmonary vein stenosis and periprocedural embolic phenomena, which has been corroborated by low complication rates in clinical studies [2].

## 5. Technology Development and Technique Modification

The use of volumetric laser heating, which is the basis of the technology utilized in LBA, was first described in 1999 as means to perform ventricular tachycardia (VT) ablation in patients with post infarction VT [14]. Using a low-power 805 nm diode laser, direct intramyocardial application was shown to produce deep subsurface lesions without visible damage to the superficial endocardium. This method was subsequently translated to performing pulmonary vein isolation and first tested in caprine animal models using a 980 nm laser with a 90 to 360° arc. In this pre-clinical study, gross pathologic and histologic examinations of cardiac muscle showed full thickness necrosis which was not associated with endocardial charring [13].

In translation of this preclinical research to the human subjects, early clinical trials using LBA technology showed a high level of success with low complication rates [8]. In the first clinical trial examining this technique, 30 patients at multiple participating centers with paroxysmal AF underwent LBA via single or double transeptal approach. The target site of ablation was the pulmonary vein—atrial juncture. Using this method, 91% of pulmonary veins were successfully isolated during the procedure. The success rate at 1 year (defined as freedom from AF) was 60%.

The findings and provider experiences from initial clinical trial data prompted subsequent changes in catheter design including a reduction in the size of the laser arc (30°, compared to 90° to 360°), as well as a switch from a non-compliant to compliant balloon to allow better apposition and contact with varying pulmonary venous diameters. The decrease in the size of the laser arc was engineered to allow for precise targeting of energy, in hopes of improving procedural PVAI success rates. In 2010, the effectiveness of the revised design was examined in a feasibility trial involving 30 patients with paroxysmal AF. In this study, 98% of the 116 pulmonary veins were successfully isolated (compared to 91% in trial involving the first-generation design). This study also featured the use of conduction gap mapping (the process of identifying islands of non-ablated tissue based on presence of electrical signals identified using a separate mapping catheter) to help address the inherent absence of ability to measure electropotentials to demonstrate real time success of ablation [15].

With this design and improved procedural technique, follow-up trials showed success rates of 89% at first attempt with a recurrence rates of only 12% and 30% for patients with paroxysmal and persistent AF respectively at 1 year, which has been validated by recently published meta-analyses [16,17].

Beyond the early success rates, studies have sought to define the AF survival-free rate at longer durations of follow up post-ablation. In one cohort study following 90 patients who underwent LBA for drug-refractory paroxysmal AF, the event recurrence-free rate of AF at 5 years was 51% (95% confidence interval (CI): 39–62%). In this study, individuals underwent repeat procedures using RFA, if there was recurrence of AF. Repeat procedures were performed in 33 individuals (37%), at a median of 499 days (181–1006) post initial ablation. In this group of individuals, the recurrence-free survival rate at 5 years (defined as recurrence of atrial fibrillation following the last ablation) was 78% [18].

## 6. Factors Influencing Success in Pulmonary Vein Isolation

Several factors have been identified which improve the likelihood of first-time acute success during PVAI using laser balloon technology, including adequate apposition of the balloon to the wall of the pulmonary vein ostia, as well as the energy source utilized. For instance, in one study comprising 30 patients, the impact of energy level utilized on successful PVAI was examined. In this study, three groups were defined: group A (5.5 and 7.0 W), group B (7.0–8.5 W), and group C (8.5 and 10.0 W). For each group, the lower wattage was applied to the posterior wall. Additionally, continuous intraluminal esophageal temperature monitoring was performed with energy delivery terminated at 38.5 °C in order to limit the likelihood of esophageal perforation and atrio-esophageal fistula formation. A follow-up upper gastrointestinal endoscopy was performed at 2 days post-ablation to detect endoscopic evidence of thermal esophageal injury.

Across the groups, the use of higher energy levels (8.5 W posteriorly and 10.0 W anteriorly) was associated with the highest rate of successful ablation (90%) without a significant difference in the detection of endoscopic evidence of thermal esophageal injury [19].

## 7. Comparisons to Other Techniques

The original rationale for the utilization of LBA for PVAI was to overcome the deficiencies that exist with RFA, particularly related to procedural time, first time and long-term success rates, and complication profiles.

In a multicenter, non-inferiority randomized control trial including 353 patients with drug-refractory AF, which compared LBA to RFA in the treatment of paroxysmal AF, the rate of freedom from recurrent episodes of symptomatic AF at 12 months was similar in both groups (61.1% in the LBA group vs. 61.7% in the control RFA group, *p* = 0.003 for non-inferiority). Additionally, rates of complications were non-inferior in the LBA group when compared to the RFA group, with the main complication of pulmonary vein stenosis being less common in the LBA group (0.0% vs. 2.9%, *p* = 0.03 for non-inferiority). In addition, the study found that after a short learning curve with LBA, experienced operators found favorable efficacy and safety compared to RF PVI [20].

To examine the rate of dormant conduction after initial PVAI, LBA was found by adenosine provocation testing (which allows detection of dormant and acute pulmonary vein reconnections), was conducted. In the randomized trial to compare acute reconnection after PVAI with laser balloon versus radiofrequency ablation (RATISOBONA trial), 50 individuals with paroxysmal AF were randomized to undergo either LBA or RFA [21]. Using adenosine provocation testing, the rate of demonstration of dormant PV connections or acute PV reconnections was found to be significantly lower in the balloon laser group (10.8%) compared to the radiofrequency ablation group (30%), showing a clear benefit of LBA for this specific outcome.

Beyond the management of paroxysmal AF, comparable clinical efficacy of LBA to RFA was seen in a multicenter European trial involving 135 patients with persistent AF (median duration 14 months) [22]. In this study, the primary endpoint of freedom from AF between 90 and 365 days was similar across both treatment groups (71.2% in the LBA, vs. 69.3% in the RFA group, *p* = 0.40), with similar complication rates across both groups.

While there are less head-to-head comparisons between cryoballoon ablation (CBA) and LBA techniques in the literature, from the limited data available, there appears comparable, if not slightly higher, procedural efficacy of LBA over CBA [23,24]. In CBA, a refrigerant is used to cool the balloon tip of the ablation catheter after inflation to produce encircling lesions at the antral level of the pulmonary veins, compared to point-by-point ablation utilized by RFA. When compared to RFA, CBA has been demonstrated to be of similar efficacy [25], with second-generation cryoballoon technology potentially reducing the rates of recurrence after 12 months [26]. Current generation cryoballoon catheters are available in two different fixed-balloon sizes, whereas the balloon of the LB catheter is adjustable in size.

The first study performing a head-to-head comparison of LBA vs. CBA for PVAI, “The Laser versus Cryo” study was published in 2013. This randomized control trial showed similar acute success rates, with similar recurrence rates of AF at 1-year post-ablation for both techniques [27]. Further comparative studies are needed to corroborate these findings, however, given the non-inferiority of both LBA and CBA to RFA using end-points of first time acute success and 1-year freedom from AF as well as complication rates, additional end-points such as procedural time, and radiation exposure might need to be examined to separate the clinical differences between these ablation techniques.

A subsequent meta-analysis published in 2018, comparing the efficacy of LBA and CBA, including 595 participants, showed equal rates of acute PVAI failure and AF recurrence at 1 year. Though not statistically significant, this meta-analysis showed that among persons with paroxysmal AF, LBA tended to have lower recurrent rates of AF at 12 months post-ablation than CBA (RR (95% CI) = 0.70 (0.47–1.04), *p* = 0.09). This, however, came at the expense of slightly longer procedural times with LBA compared to CBA [23].

Attention should be paid to the safety profile of LBA and how it compares to other established AF ablation techniques such as RFA and CBA. In the phase 3 trial “Chronic effective in Treating PAF as Demonstrated by no AF Recurrences After the Banking Period and During the 12-month Follow-up Period”, of the 72 enrolled participants, serious adverse events occurred in 14% of participants. The most common adverse events included pericardial tamponade (5.6%), pericardial effusion (4.2%), air embolism (2.8%), and phrenic nerve injury (1.4%) [28]. When compared to RFA and CBA techniques, LBA has been found to have similar safety profiles in multicenter clinical studies [22,27].

## 8. Technique Advances and Emerging Technologies

**Figure 4:**
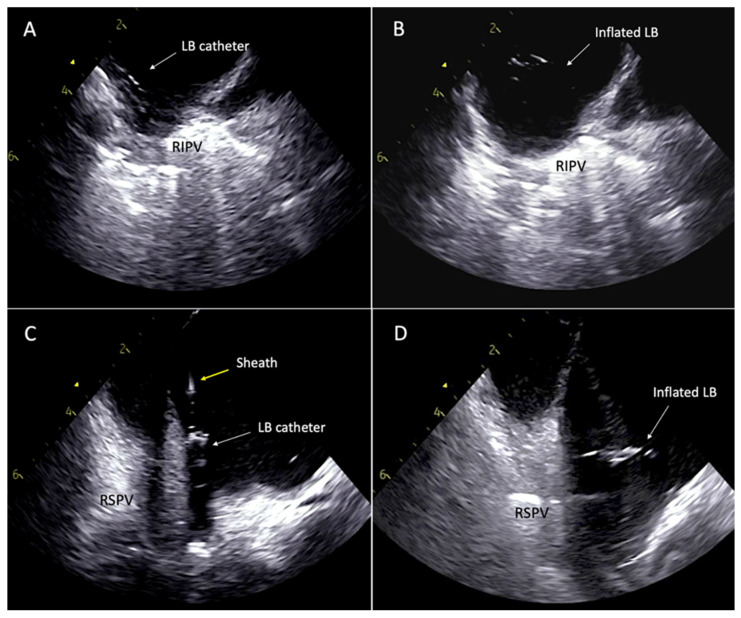
LB: laser balloon; RIPV: right inferior pulmonary vein; RSPV: right superior pulmonary vein; ICE: Intracardiac echocardiography. Low fluoroscopy ICE-guided approach for LB ablation. (**A**,**B**) LB catheter directed into RIPV using ICE guidance and inflated prior to ablation. (**C**,**D**) RSPV cannulated under ICE and inflated into antral position.

One area of development in LBA PVAI has aimed to reduce the procedural fluoroscopy time, which was high in early studies, though similar to RFA according to one large study (14 ± 9 min vs. 11 ± 9 min *p* = 0.08) [22]. A recent study using an ICE catheter to create a 3-D anatomic shell of the LA and guide pulmonary vein cannulation and LB catheter positioning (Figure 4) was shown to be effective at reducing the fluoroscopic time by 95% when compared to the conventional fluoroscopy methods for LBA (1.7 ± 1.4 vs. 16.9 ± 5.9 min; *p* < 0.001) [29]. This novel approach was not associated with increased intra-procedural time or increased complication rates. Perhaps with time this novel integration of 3-D ICE into procedural workflows may become standard practice when performing LBA to reduce operator and patient radiation exposure.

Recently a third generation of the device (HeartLight X3 Endoscopic Ablation System^®^, CardioFocus, Inc.) has been approved by the U.S Food and Drug Administration and features a rapid mode which allows continuous, controlled energy delivery which allows for single-shot, high-speed circumferential PVAI (Videos S1–S3) [30]. Initial data presented at the 2019 Heart Rhythm Society scientific session suggested a reduction in procedural time by more than 90 min with procedures able to be completed within one hour. This was associated with faster acute PVAI times (said to be 7 times faster than the first-generation device) with reduced fluoroscopy times. The overall procedural safety was comparable to earlier generations of the device, with potentially lower occurrence of phrenic nerve injury [31]. In a prospective study comparing the 2nd (LB2) and 3rd generation (LB3) LBA systems, Heeger et al. [32] demonstrated significant reductions in total procedure time (LB2: 91 (IQR: 86, 105) min vs. LB3: (77 (IQR: 68, 87) min; *p* < 0.001) and laser ablation time (LB2: 1920 (IQR: 1765, 2193) s vs. LB3: 1077 (896, 1165) s; *p* < 0.000001) without difference in occurrence of major adverse events (LB2: 1/15 (6.7%) vs. LB3: 1/15 (6.7%); *p* = 0.999). Further, while pulmonary vein diameter, shape, and branching can adversely affect AF-free survival with CB PVI ablation [33,34], PV anatomy does not seem to hold similar negative impact on clinical outcomes for LBA, particularly as the introduction of LB2 system, because of the compliant, adjustable sized balloon design which confirms readily to most PV anatomy [35].

Beyond laser balloon ablation, other novel techniques for PVAI are being developed but they remain in varying investigative stages. One such technique is High-Power and Short-Duration (HP-SD) ablation, which uses very high-power short-duration pulse energy sources during PVAI. The aim of this device design is to reduce the time taken to perform PVAI ablations [36]. Given the infancy of this technology, a comparison of this technique to LBA has not yet been described.

## 9. Conclusions

There are many applications for the use of lasers in medicine. The role of this energy source for catheter-based pulmonary vein isolation ablation (PVAI) is evolving with clinical trials showing comparable outcomes (first time success and 1-year freedom from atrial fibrillation), and similar (perhaps better) favorable complication profiles when compared to radiofrequency ablation (RFA) and cryoballoon ablation (CBA). With further improvements in this technique, such as with the use of low fluoroscopy protocols, rapid-mode ablation, as well as the development of newer generations of the catheter systems, we anticipate that the use of the modality will become more widespread and commonplace in clinical practice. In addition, the success of PVAI as measured by freedom from AF at 5 years, will likely improve as well.

## Supplementary Materials

The following are available online at https://www.mdpi.com/2072-666X/12/2/188/s1, Video S1: Laser ablation (endoscopic view) of atrium of large right superior pulmonary vein in continuous rapid mode using 3rd generation laser balloon system, Video S2: Laser ablation (endoscopic view) of posterior wall behind right inferior and middle pulmonary veins, Video S3: Stability of laser ablation (endoscopic view) in continuous rapid ablation mode of the anterior antrum of the right superior pulmonary vein during phrenic nerve pacing.
